# Complex Collagen Fiber and Membrane Morphologies of the Whole Porcine Aortic Valve

**DOI:** 10.1371/journal.pone.0086087

**Published:** 2014-01-21

**Authors:** Christopher A. Rock, Lin Han, Todd C. Doehring

**Affiliations:** School of Biomedical Engineering, Science and Health Systems, Drexel University, Philadelphia, Pennsylvania, United States of America; Brigham and Women’s Hospital, Harvard Medical School, United States of America

## Abstract

**Objectives:**

Replacement aortic valves endeavor to mimic native valve function at the organ, tissue, and in the case of bioprosthetic valves, the cellular levels. There is a wealth of information about valve macro and micro structure; however, there presently is limited information on the morphology of the whole valve fiber architecture. The objective of this study was to provide qualitative and quantitative analyses of whole valve and leaflet fiber bundle branching patterns using a novel imaging system.

**Methods:**

We developed a custom automated microscope system with motor and imaging control. Whole leaflets (n = 25) were imaged at high resolution (e.g. 30,000×20,000 pixels) using elliptically polarized light to enhance contrast between structures without the need for staining or other methods. Key morphologies such as fiber bundle size and branching were measured for analyses.

**Results:**

The left coronary leaflet displayed large asymmetry in fiber bundle organization relative to the right coronary and non-coronary leaflets. We observed and analyzed three main patterns of fiber branching; tree-like, fan-like, and pinnate structures. High resolution images and quantitative metrics are presented such as fiber bundle sizes, positions, and branching morphological parameters.

**Significance:**

To our knowledge there are currently no high resolution images of whole fresh leaflets available in the literature. The images of fiber/membrane structures and analyses presented here could be highly valuable for improving the design and development of more advanced bioprosthetic and/or bio-mimetic synthetic valve replacements.

## Introduction

Heart valves are specialized structures that prevent the backflow of blood into the chambers of the heart. This function can be undermined by diseases such as calcification or congenital defect. In 2009, over 139,000 medical procedures were performed in the US related to heart valves, of which 89,000 were in patients 65+ years old [1]. This presents a growing concern considering the rising elderly population. Two-thirds of heart valve related hospital discharges and mortalities involve the aortic valve [1], hence the aortic valve is of particular interest. If conservative treatment such as valve repair cannot sufficiently restore a valve’s function, a replacement valve made of either synthetic or biological material will be installed. While the current prosthetic valves have undeniable medical value, there are drawbacks in terms of durability and biocompatibility which underscore the need for better understanding of valve ‘mesostructure’ (i.e. fiber bundle and membrane structures) to potentially enable a more modern tissue engineered repair/solution [2], [3].

**Figure 1:**
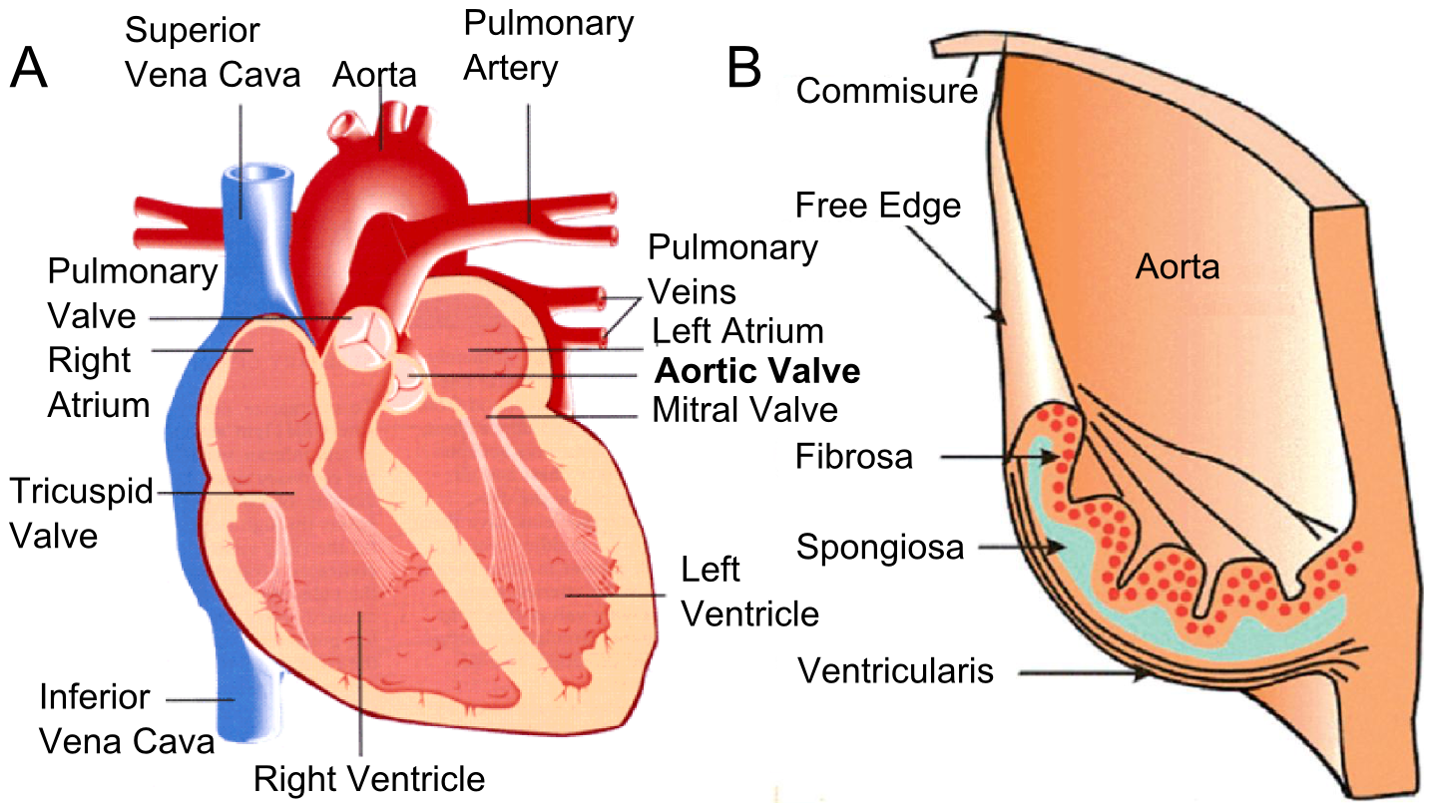
Schematics of heart valve anatomy. (**A**) The arrangement of the valves in the heart. (**B**) The structures and layers of an aortic valve leaflet.

The aortic valve is an arrangement of 3 leaflets identified by the presence of 2 coronary artery ostioles yielding a left coronary, a right coronary, and a non-coronary leaflet. Current anatomic texts simply describe the leaflet as comprised of three layers (Fig. 1). On the aortic side, the fibrosa is an arrangement of collagen sheets and large collagen fiber bundles. On the ventricular side, there is the thin ventricularus layer. Between these outer layers is the spongiosa, a layer rich in proteoglycans. The layered structure (taken largely from inspection and analysis of histological specimens) is described as an adaptation to the multiple functional requirements for frequent flexion duty cycle, durability, high shear compliance, and high pressures of aortic hemodynamics [4], [5], [6]. This idealized description is useful as a basic representation of valve anatomy. However, it is well known that valve material and biomechanical properties can vary from leaflet to leaflet and even within an individual leaflet [7], [8], [9].

Few studies have visualized and quantified the true fiber/membrane anatomical morphology at whole-leaflet scales [10]. The organization of larger leaflet fiber/membrane structures; existing on a scale between the macroscopic whole leaflet and the microscopic individual collagen fibers is referred to by us as the valve “mesostructure”. Initial works exploring the aortic valve mesostructure has suggested that the aortic leaflet mechanical properties are related to conformation. For example, the asymmetric arrangement of fiber bundles in the left coronary leaflet may be related to optimization of blood flow hemodynamics and contribute its anisometric mechanics [10], [11]. Previously, using the polarized light microscopy, we highlighted some key differences between three leaflets, including their width, height and fiber bundle count. However, due to the imaging system limitations of spatial resolution [10], there was no detailed, mesoscale quantification of the leaflet structures, including the fiber bundle arrangements, membrane layout and the fiber-membrane anchoring. Knowledge of the mesostructural features, e.g., the fiber/membrane morphology, are critical for understanding the hierarchical design and function of the leaflets that can be used for biomedical applications such as prosthetic design. is critical to understanding these structure-function relationships.

The overall objective of this work was to understand the key features of the whole porcine aortic valve leaflets at the sub-micrometer mesoscale using a custom-built autofocusing microscope with elliptically-polarized light capability. Due to the extreme difficulty in obtaining normal, disease-free human heart valves and the similarity between porcine and human valves have similar structure [12], we used porcine whole leaflets as the model system here. In this study, we quantified and compared sub-micrometer scale structure of the fiber bundles and membranes across the 3 leaflets, and between the left and right “sides” of each leaflet. We identified new features of the fiber bundles and their branching patterns that were not observed previously. Both qualitative and quantitative analyses were carried out to understand the heterogeneity at both the whole-organ scales between the three leaflets and at the mesmoscale, fiber-bundle levels. The long term application of this study is to improve understanding of the complex structural basis for the valve’s evolved biomechanical properties. The knowledge obtained from this study This can be applied to models that more accurately depict valve dynamics and may guide the development of more anatomically accurate tissue engineered heart valves.

## Materials and Methods

### Specimen Preparation

**Figure 2:**
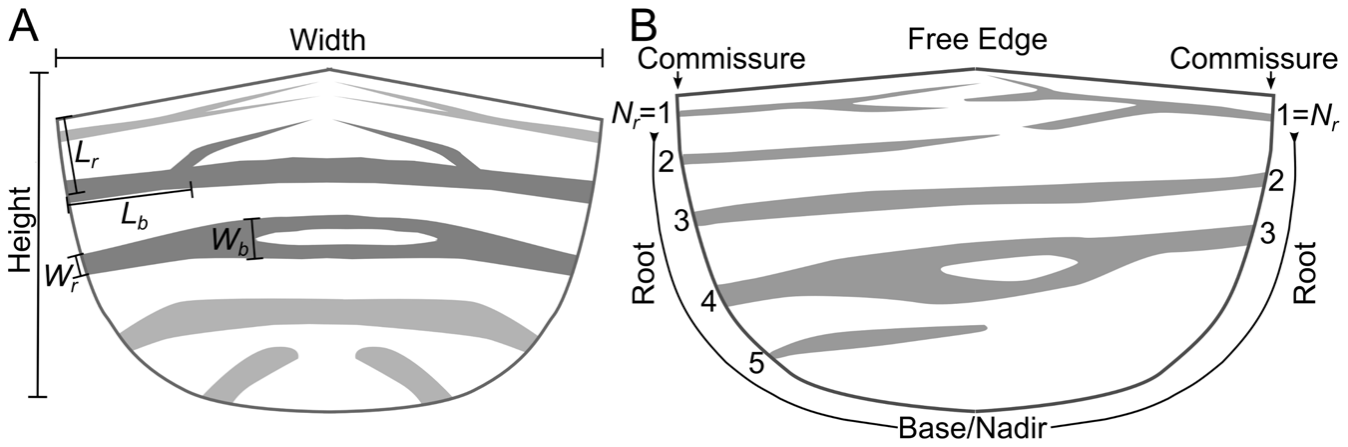
Schematics of a leaflet illustrating the structural parameters used to quantitatively describe the leaflet mesostructure, including 1) the length from the free edge along the root, *L_r_*, 2) the distance along a fiber bundle to the first branching, *L_b_*, 3) the width of the fiber bundle at the root, *W_r_*, 4) the width of the fiber bundle before the first branching, *W_b_*, and 5) the position of the fiber bundle from the free edge along the root, *N_r_*.

Fresh hearts from six month old pigs were acquired from a local abattoir and kept in ice during transport. The aortic valve was dissected out along with the aortic root from the heart. Each valve was cut through the root between the leaflets opening the valve to expose the leaflets. The three individual leaflets, left, non-coronory and right were carefully separated from the root using surgical scissors. The width of each leaflet was measured using a caliper in phosphate buffered saline (PBS) as the the distance from left to right commissures (Fig. 2A). The height of each leaflet was measured as the distance from apex of the free edge to base of the leaflet) (Fig. 2A). All of the leaflets were used within 2 hours of extraction.

### Imaging Procedure

Images were acquired using a digital camera (model PL-A686 PixeLINK Inc, http://www.pixelink.com) mounted to an Olympus microscope (model BX50 Olympus Inc.) equipped with polarizing filters and a ¼λ wave plate. The microscope was retrofitted with motors for fully automated stage control via a connected PC computer and custom software/hardware developed in-house. Leaflets were placed on a glass slide with the aortic side (fibrosa layer) on top. We scanned leaflets from one heart with each layer on top and this produced negligible differences in the resulting images (Figure S1). In order to expose more of the structure to the camera and prevent the specimen from drying during image acquisition, as second glass slide was placed on top of the leaflet with additional PBS added to retain the hydration and reduce the formation of air. This second slide provides a slight pressure to flatten the leaflet, At 4× magnification, the camera field of view was 2.59 mm×1.90 mm at a resolution of 1160 pixels/mm (0.862 µm/pixel).

During imaging, each leaflet was scanned left to right, row by row using an automated stage. Each image was focused using a novel algorithm that automatically adjusted for the best whole-image focus via a stepper-motor controlled z-axis. The autofocusing algorithm used edge detection and pixel counting to determine maximum sharpness (minimizing blur). The row-by-row scan produced a rectangular mosaic of images with 10% vertical and horizontal overlap between adjacent images. An elliptical light polarization was applied during imaging by rotating the polarizing filter 12° clockwise from a black background. due to the orientation changes in the birefringent aotic valve collagen fibers. Because collagen, the major protein of the aortic valve leaflet, is birefringent, this polarized light produced color contrast on this translucent tissue as a result of collagen orientation changes induced light phase shifts [13]. We were thus able to delineate the collagen orientation and thickness without employing stains or markers from the resulted images with a dark violet background, a blue-green membrane, and red-yellow fiber bundles. These images were acquired via a custom designed/programmed GUI.

### Quantification of Aortic Leaflet Mesostructure

The image arrays were imported into the free Microsoft Image Composite Editor (ICE, Microsoft Inc.) for stitching using the planar scan modality. Accuracy of mosaic reconstruction was confirmed by visual inspection. The resulting composite images have a resolution on the order of 30,000×15,000 pixels.

Images of a total of twenty-five full leaflets were produced, including 8 left coronary, 8 non-coronary, and 9 right coronary leaflets. The image resolution was reduced by 2× before importing the images into GNU Image Manipulation Program (GIMP, http://www.gimp.org/) for visual inspection, general observations, and to calculate metrics of the fiber bundles. Decimation by 2 did not affect following measurements or statistics, and greatly reduced computer memory requirements and analysis time.

A list of quantitative parameters were measured to quantify the mesostructure of each fiber bundle using on-screen analysis tools via GIMP, as illustrated in Fig. 2. These parameters include 1) the distance from the root origin of the fiber bundle to the free edge of the leaflet, *L_r_*, 2) the distance from the root origin of the fiber bundle to the first major branching, *L_b_*, 3) the width of the fiber bundle at the root, *W_r_*, 4) the width of the fiber bundle before the first major branching, *W_b_*, and 5) the fiber bundles’ position along the root from the free edge, *N_r_*. Data were imported into the R software (http://www.r-project.org/) and Matlab (Matlab2010a, The Mathworks, Inc., Natick, Massachusetts) for statistical analysis.

### Statistical Analyses

To investigate the structural variations between these leaflets, we used analysis of variance (ANOVA) tests to compare the parameters from each leaflet. One-way ANOVA was used to compare leaflet height and width across the 3 leaflets and ratio of left versus right fiber bundle counts between each leaflet. We also used one-way ANOVA to study the effects of *N_r_*, side of leaflet (left or right), and leaflet (left coronary, right coronary, or non-coronary) on *W_r_*, *L_b_* and *W_b_*/*W_r_*. Least squares linear regression was used to test the correlations between: *N_r_* and *L_r_*, *L_b_* and *W_r_*, *W_b_* and *W_r_*. In all the statistical tests, a *p*-value of less than 0.05 was taken as statistically significant.

## Results

**Figure 3:**
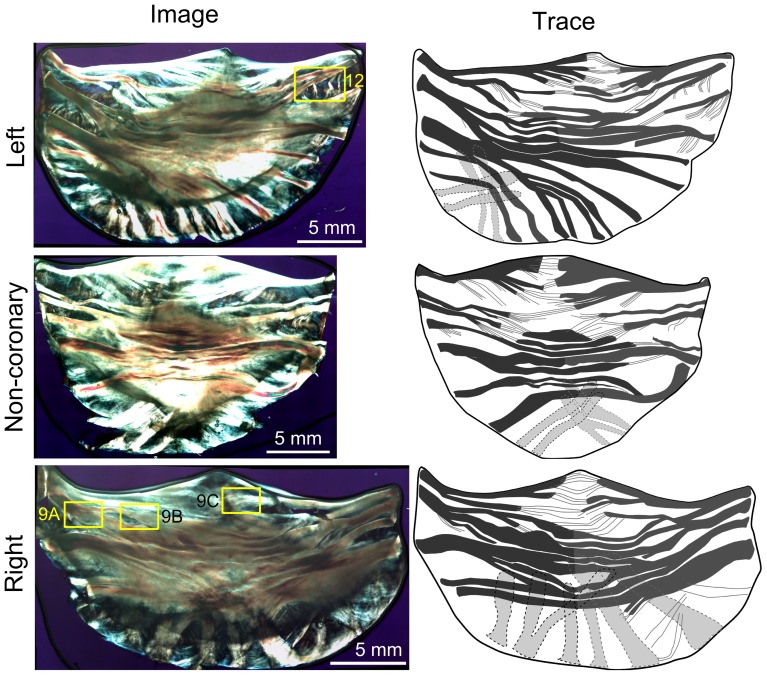
Polarized light images of three representative aortic valve leaflets. Images of three representative leaflets (left) along with traces of the major fiber bundles (right) to clarify primary structures of the leaflets. Fiber bundles appear yellow-orange and membranes appear blue as a result of the use of transmitted elliptically polarized light imaging. Clear differences in the fiber bundle arrangement were shown in the three leaflets. The left leaflet was the most asymmetric, while the non-coronary and right leaflets were more symmetric. Fiber bundles were most distinct at the edge (root) and branched or spread out towards the belly of the leaflet. Overall, fiber bundles appeared to present a cross-hatched, overlapping structure with thin connecting membranes. The yellow boxes identify the regions presented in Figures 8 and 12.

Full scale, elliptically polarized light microscope images were acquired for a total 25 leaflets. Representative samples for each of the three leaflets (left, right, and non-coronary) are shown for comparison along with tracings of the major fiber bundles to better illustrate the major structures (Fig. 3). Clearly visible under elliptically polarized light are extensive and complex arrangements of fiber bundles and connecting membranes, i.e., the mesostructure of the leaflet.

**Figure 4:**
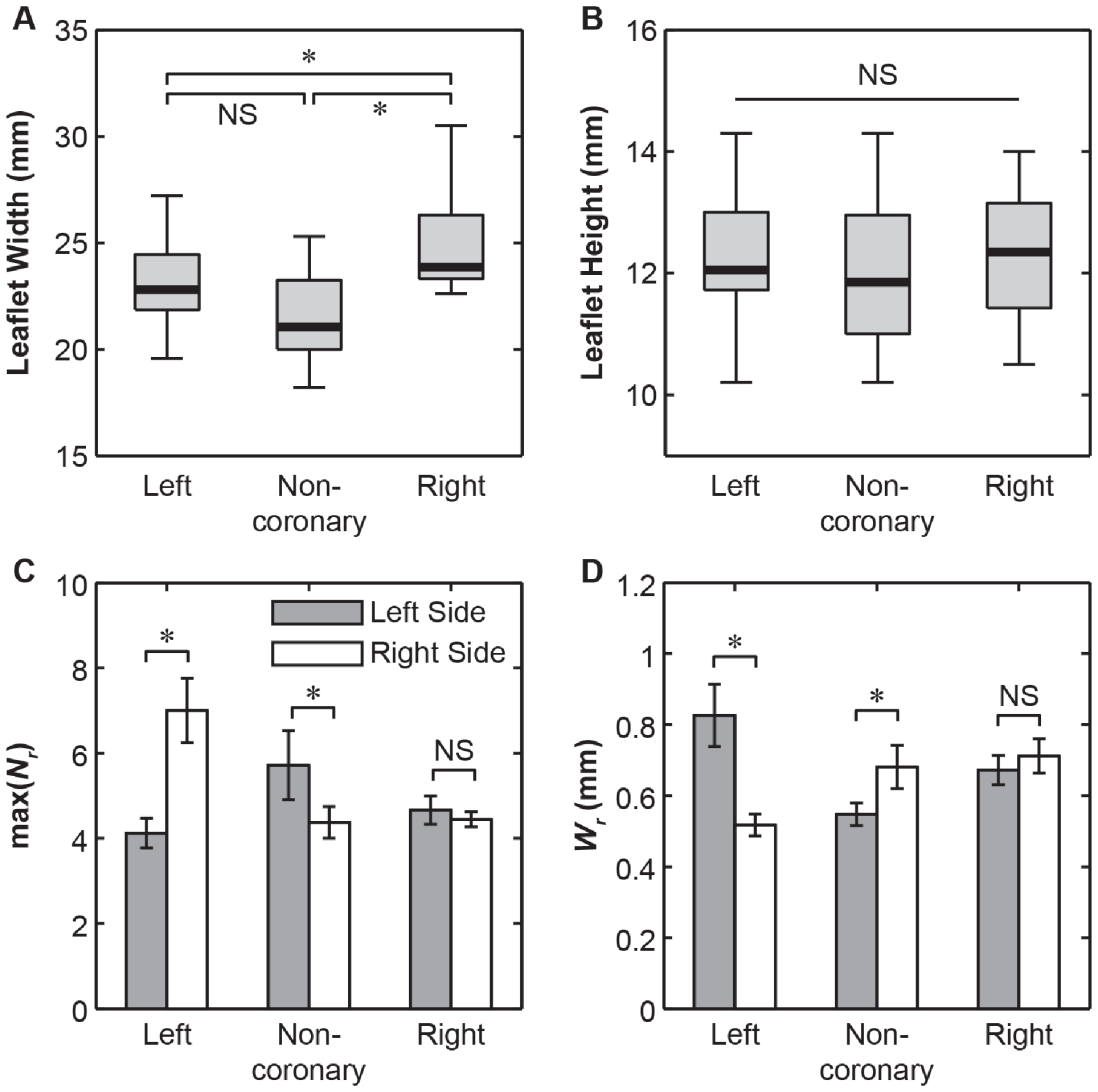
Figures of whole leaflet properties. (**A**) Boxplot of aortic leaflet widths. The leaflet widths are significantly different with the right coronary leaflets being the widest, followed by the left coronary leaflets and the non-coronary leaflets being the least wide (*: *p*<0.001. via one-way ANOVA, *n* = 16). (**B**) Boxplot of aortic leaflet heights, where no significant difference was found between the heights of the leaflets (*p*>0.05 via one-way ANOVA). (**C**) Bar graphs of average fiber bundle counts (max of *N_r_*) for each side of each leaflet (mean±SEM, *: *p*<0.05 via student’s *t*-test between each side of the coronary, *n* = 7). (**D**) Bar graphs of average fiber bundle width at the root (*W_r_*) for each side of each leaflet. (mean±SEM, *: *p*<0.01 via student’s *t*-test between each side of the leaflet, *n* = 33).

**Figure 5:**
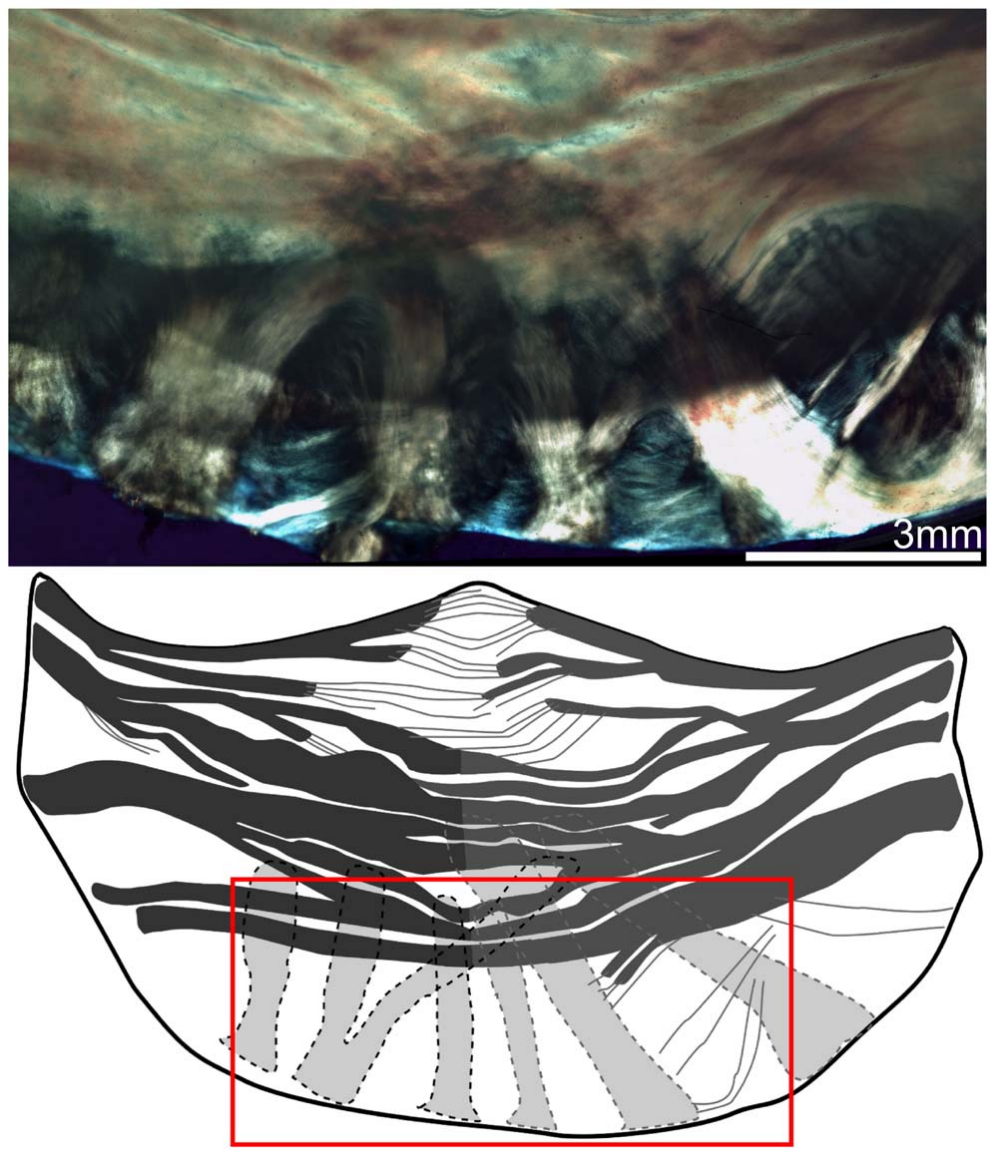
Polarized light image of the right aortic leaflet with trace highlighting the underlying fiber bundles (shown in light grey in the trace) that travel upwards from the nadir of the leaflet.

**Figure 6:**
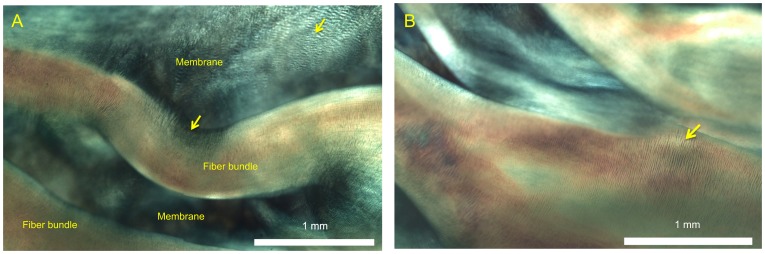
Polarized light images of aortic valve mesostructure. (**A**) Image of typical fiber bundle (orange) and membrane (blue, blue-green) structures showing the crimping patterns typical of collagen (arrows) and highly parallel fibril organization in the fiber bundles. (**B**) Image of complex overlapping fiber-membrane structures showing crimping patterns typical of collagen (arrow), as well as the fiber bundles branching and extending inwards from the root of the leaflet, interconnecting with membranes with fan-like and pinnate structures.

Whole valve-level morphological differences between these three leaflets were clearly visible using this imaging technique. We observed the right coronary leaflets to be the widest, followed by the left coronary leaflets, and the non-coronary leaflets are the narrowest (*p*<0.001, Fig. 4A). On the other hand, there were no significant variations regarding their root-to-coapting heights (*p*>.05, Fig. 4B). The left coronary leaflet was found to be highly asymmetric, as reflected by the unequal number of total fiber bundles max(*N_r_*) on each side (*p*<0.001, Fig. 4C, Figure S2). In comparison, this structural asymmetry is much less substantial for the non-coronary leaflets, and absent for the right coronary ones (Fig. 4C). Each side has on average 5.5 fiber bundles so all figures will analyze only the first 5 when plotting against *N_r_*. Within each leaflet, the fiber bundles are mostly aligned transversely, nearly parallel to the free edge, except for those at the base, where they take a more radial-like direction (Fig. 5). At the nadir, some bundles were observed to travel underneath the others and cross over each other. For the transverse fiber bundles, they extend inward from the root of both sides, tapering and branching as they traversed to the middle of the leaflet. Each fiber bundle is composed of well aligned, highly parallel collagenous fibers, similar to other fibrous connective tissues such as tendon [14]. Images also revealed crimp patterns, a common feature for collagen fibers [10] (Fig. 6).

**Figure 7:**
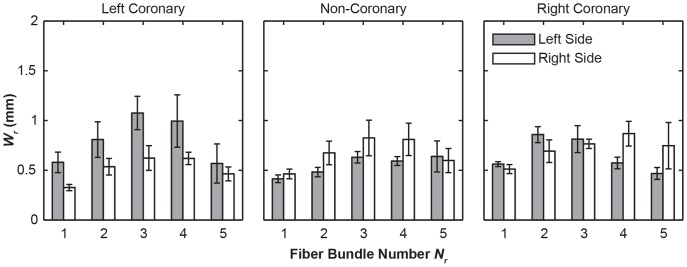
Bar graphs of average fiber bundle root witdth (*W_r_*) as a function of position from the free edge (*N_r_*). The fiber bundles toward the center of the leaflet (*N_r_* = 3,4) tended to be the widest with the addition of the second fiber bundle on the right coronary leaflet. Error bars indicate the standard error.

**Figure 8:**
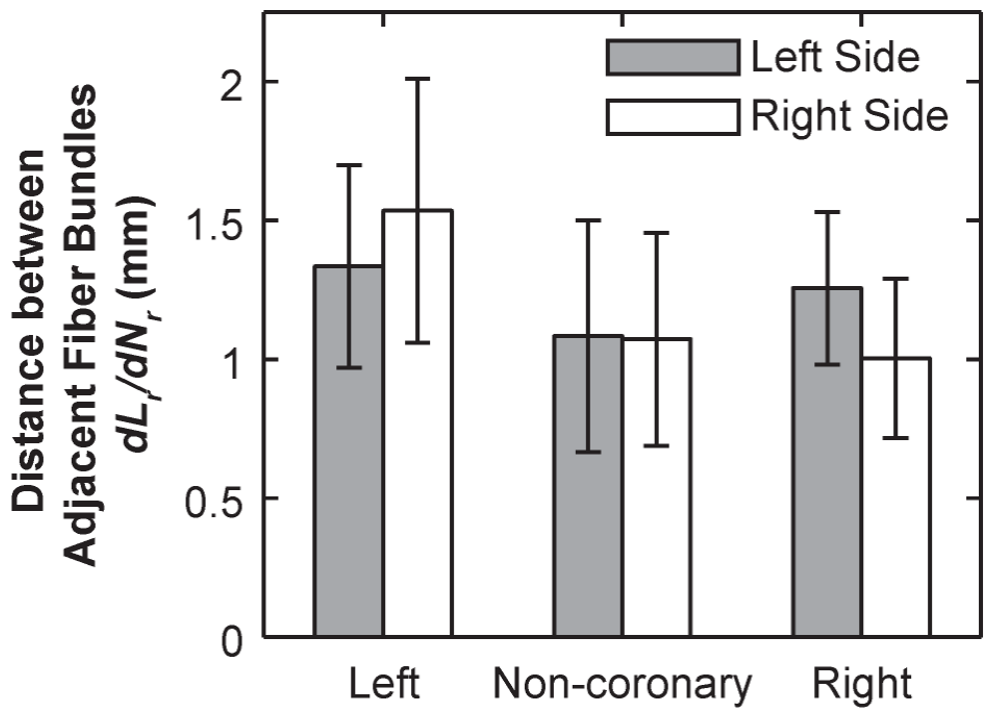
Bar graph of average distance between adjacent fiber bundles on each side (mean ±95% confidence intervals), calculated by least squared linear regression of *L_r_* over *N_r_* on each side of each leaflet (*R* ^2^>0.81 for all regressions).

Within each leaflet, these fiber bundles display significant variations in its root width, *W_r_*. The fiber bundles ranged from 0.174 mm to 2.7 mm (*W_r_*) in width at the root, whereas the widest bundles were found near center of each leaflet (*N_r_* = 3 or 4, except *N_r_* = 2 for the left side of right coronary, Fig. 7). The values of *W_r_*, are significantly greater on the left side for the left coronary leaflet, on the right side for the non-coronary leaflet, and no difference for the right coronary leaflet, as shown in (*p*<0.0001, Fig. 4C). Overall, the distance between fiber bundles averaged 1.12 mm from center to center at the root. This varied across the leaflets with the left coronary leaflet having larger gaps between fiber bundles relative to the non-coronary and right coronary leaflets (*p*<0.0001, Fig. 8).

**Figure 9:**
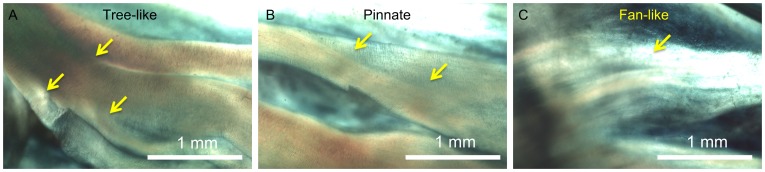
Higher resolution polarized light images of fiber bundle branching patterns from Fig. 3. Three branching mechanisms were shown: (**A**)Tree-like fiber bundle branching. (**B**) Pinnate fiber branching. (**C**) Fan-like fiber branching.

**Figure 10:**
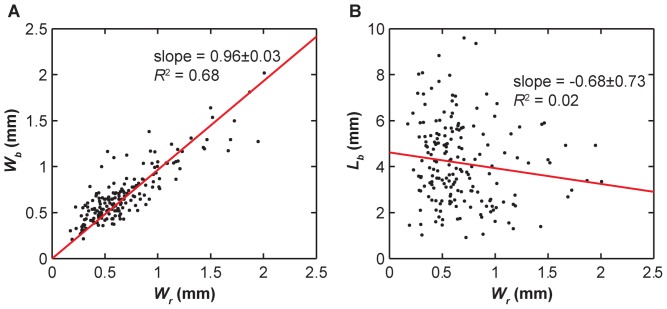
Linear regression of branching measurements. (**A**) Graph of the fiber bundle width at the first branch point (*W_b_*) relative to the width at the root (*W_r_*). The trendline has an R^2^ value of.68 suggesting some relationship between *W_b_* and *W_r_*. The slope of the trendline is close to 1 suggesting that the fiber bundles do not widen before the first branch point. (**B**) Graph of the length along fiber bundle to the first branch point (*L_b_*) relative to the width at the root (*W_r_*). The trendline has an R^2^ value of.02 suggesting no correspondence.

**Figure 11:**
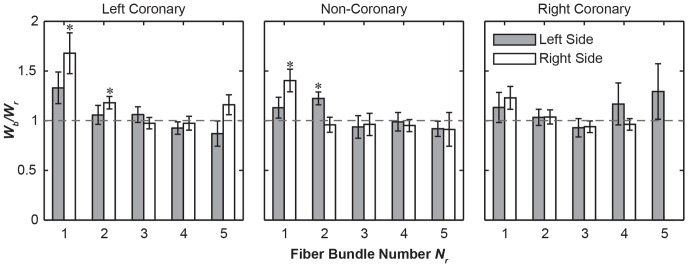
Bar graphs of the relative widening (*W_b_*/*W_r_*) as a function of fiber bundle position position from the free edge (*N_r_*) (mean ± SEM). (*: *p*<0.05 via student’s *t*-test).

In all of these three leaflets, three distinctive types of fiber bundle branching patterns were observed : 1) tree-like branching, in which each fiber bundle split into 2 or more bundles of similar form (Fig. 9A), 2) fan-like branching, where the fibrils of the bundle would start to spread out but maintained some of their parallel organization like the bristles of a brush (Fig. 9B), and 3) pinnate branching, where the bundle completely separated into its constituent fibrils embedding into the underlying membrane (Fig. 9C). Among these three modes, the tree-like branching was the most frequent mode, except for those at the nadir, which generally did not branch out. The pinnate branching mode occurred exclusively near the free edge of the leaflet (bundles of *N_r_* = 1 or 2). The fiber bundle width at the branch point (*W_b_*) remained unchanged relative to the width at the root (*W_r_*) (Fig. 10A). This appears to hold true when comparing leaflets (*p*>.05) and left versus right side (*p*>.05). However, the fiber bundles widened significantly in the first fiber bundle (*p*<0.0001, Fig. 11). The point of branching of a fiber (*L_b_*) is related to its position (*N_r_*) along the leaflet (*p*<0.05) but is not significantly related to leaflet (*p*>.05), side (*p*>.05), or fiber bundle width (Fig. 10B, Figure S3).

**Figure 12:**
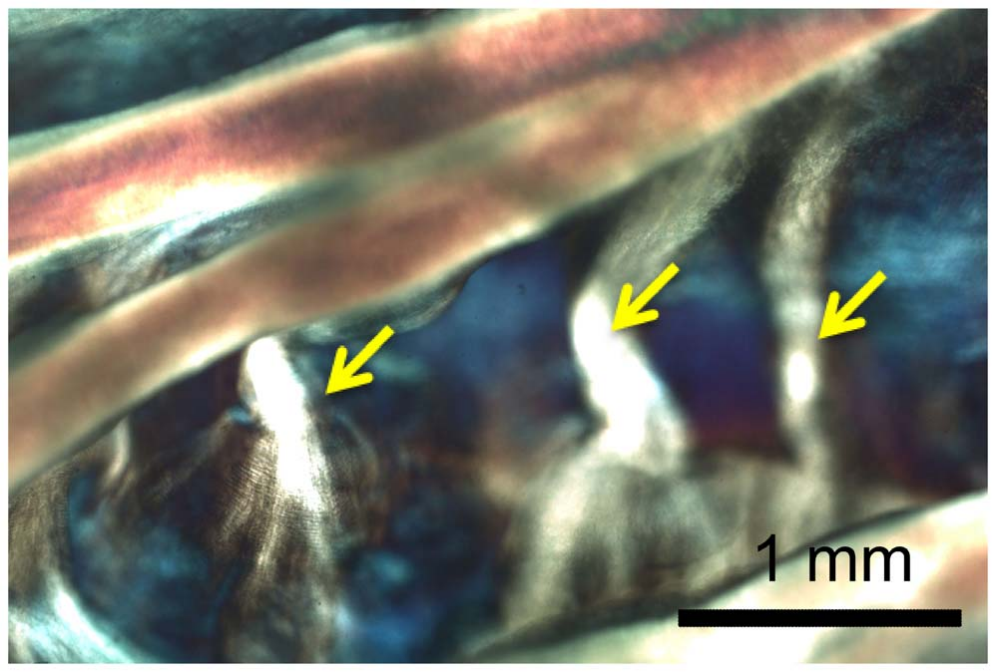
Higher resolution optical image of the left coronary leaflet showing the anchoring structures of fiber bundles, with fibrils branching off into the connecting membrane.

We also revealed more structural details of the membranes. The membrane was shown to contain very thin ‘sheets’ of collagen again with plainly visible crimp patterns (Fig. 6A). Often two or more sheets appeared to ‘overlap’. In comparison to the highly ordered collagen alignment in the fiber bundles, the membranes displayed a less organized collagen alignment, with multiple overlapping regions of locally parallel fibril bundles (Fig. 6B). The membranes appeared to be larger without fiber bundles in the regions close to the free edge, and smaller near the belly. The membranes and fiber bundles appeared in in many regions to be connected by an ‘anchoring’ structure (Fig. 12) consisting of collagen fibers that spread from the bundle near the root and extend into the underlying membrane in a manner similar to the fan-like pattern. These structures were observed mostly in the fiber bundles away from the free edge (*N_r_* = 2, 3 and 4). Unlike the branching mechanism, anchoring did not appear to alter the thickness of the main fiber bundle.

## Discussion

The overall goal of this study was to provide a quantitative evaluation of meso-scale fiber and membrane structure of the aortic valve cusp leaflets, which can contribute to the understanding of the structure-function relationships of the aortic valve. To achieve this goal, we implemented a novel computer controlled, automatically focusing imaging system at micron scale resolution, for the purposes of large scale imaging (up to 60,000×40,000 pixels) of fresh (and hence thick) specimens in fluid. Using this approach, we carried out quantitative, detailed structural characterization on individual fiber bundles and their associated membranes. These studies thus provided the quantitative evidences on the complex collagenous structure of the aortic valve that serves as the basis for understanding its loading environment, tissue function and for bioprosthesis designs.

### 1. Structure Variation between Different Cusps

Our previous work has identified the structural variations between three valve cusps, including the cusp areas, average bundle diameters and fiber bundle count [10]. In this study, with the improved imaging technique, we further investigated the cusp-to-cusp structural variations that could provide insights into their different mechanical environments and tissue functions. For example, significant variation in the leaflet width (Fig. 4A) could be adaptations for the dynamics of the aortic root during the cardiac cycle [11]. The high asymmetry in the left coronary cusp, including total number of fiber bundles (max(*N_r_*), Fig. 4C), fiber root diameter *W_r_* (Fig. 4D) could be correlated with the assymetric tensile and compressive stress executed on the tissue. There is an inverse correlation with the total number of fiber bundles, max(*N_r_*), on each side of the leaflet and the size of the fiber bundles, *W_r_* (Fig. 4C,D). This correlation is most pronounced in the asymmetric organization of the left coronary leaflet (Fig. 4C,D), which is suggested to be designed in a manner to accommodate the higher compliance of the left coronary leaflet [8]. In comparison, this asymmetric effect is less substantial for the non-coronary leaflet, and insignificant for the right coronary leaflet (Fig. 4C,D). The space between fiber bundles was also greatest in the left coronary leaflet which could increase the overall flexibility of the whole leaflet (Fig. 8). Taken together, these differences provide detailed information into how bioprosthesis and valve implantation can be designed to emulate the tissue-level heterogeneity of the valve.

### 2. Mesostructure of Cusp Fiber Bundles and Membrances

This study is the first kind of detailed, quantitative investigation on the mesostructure of the aortic valve fiber bundles and membranes. The results of this study deviate from the commonly used, trilayered model of the aortic valve structures [5], [15]. As our previous work [10] acknowledged the complexity in the structure, this study further investigated the sub-tissue-level features of this high complexity, multilayering and asymmetry of the cusps, which likely reflect the optimization of its tissue function.

In particular, we found that the fiber bundle diameter is largest in the middle of the leaflet, decreasing as bundles are closer the free edge or nadir of the fixed end (Fig. 7). This is consistent with previous observation that individual fibril diameter increases traversing from the fixed edge of the leaflet to the free edge [7], and the fiber bundles roughly equidistant from the lowest point of the root and the free edge are the thickest [10]. These results together suggested that the fiber bundle diameter is dependent both on the number of fibrils and their diameter. This higher *W_r_* and smaller membrane regions may be an adaptation to the peak stress and strain occurring in the belly of the leaflet [11]. It is also correlated with the presence of additional anchoring structures supporting the bundles in the high strain regions. For example, the pinnate structure (Fig. 9) may facilitate strain distribution from the fiber bundle to belly while permitting large flexion compliance.

In addition, the fiber bundles were found to maintain a consistent thickness as they travel from the root (*W_r_*) to their first branch (*W_b_*) (Figs. 10A and 11). This feature is beneficial in homogenizing the stresses through the fiber bundles and preventing loading spikes. One exception is that we always observed widening at the branching point for the first bundle for the left and non-coronary leaflets (*N_r_* = 1, Fig. 11A, B), which is likely a result of fibril dispersion in the branching patterns other than the “tree-like” mode (Fig. 9). In addition, the distance from the root to the first branching point (*L_b_*) was found to be independent of the root thickness *W_r_*, (Fig. 10B) the leaflet, and the side of the leaflet (*p*>0.05, data not shown).

Besides the variation in each fiber bundles, we also identified the ‘anchoring structures’ that appear to connect the fiber bundles to their adjacent membranes, including the ‘tree-like’, ‘fan-like’ and pinnate fiber branching patterns (Fig. 9). While tree-like branching was reported previously [10], [16], [17], to our knowledge, it is the first time that the distinctive fan-like and pinnate branching structures were reported. Interestingly, over the 25 leaflets tested here, we did not observe the “fractal” branching pattern that was described previously [16].

Overall, these variations in the fiber bundle morphology indicated that there are many ‘pathways’ to a functionally successful valve with respect to specimen-specific leaflet mesostructure. These results also indicated that mechanics (hemodynamics) may play a significant role in valve development. It is likely that genetics provides the ‘initial conditions’ for the valve’s basic morphology, and then the valve develops and remodels over time *via* biomechanical influences that ‘fine-tune’ a final optimal structure for the individual. Further studies on the genetic conditions and biomechanical environments are required to fully understand the origins of this mesoscale heterogeneity.

### 3. Autofocusing Imaging Technique

A key aspect of this custom imaging system was the implementation of a novel auto-focusing algorithm. Most current auto-focus systems employ a contrast based method, using either center focusing or spot-arrays. Previous contrast method often produced inconsistent results and out-of-focus images for specimens with large and heterogeneous thicknesses. In this study, we applied an edge detection based algorithm which uses the entire image to achieve an optimal overall focus in a consistent fashion. The net effect is the substantial increase in image resolution (∼ 0.86 µm/pixel) over our previous publication (∼ 14.8 µm/pixel) [10]. This improved design allowed detailed quantification of the heterogeneity of these leaflets at both whole tissue and fiber bundle levels presented here. Besides its application to the aortic valves, this method also holds great potential for studying the mesoscale features of other collagen-based connective tissues, such as tendon.

### 4. Limitations and Outlook

We identified several technical limitations of the method that can be further improved. Firstly, there is no automatic modulation of the exposure in the software, which leads to over-illumination of relatively thinner regions, and lack of illumination in thicker regions. Secondly, the stitching program sometimes resulted in minor misalignment. However, as we found most images are well-aligned, we expected these technical issues to have minimal effect on the data or any conclusion in this study. We are currently working on improving the high-dynamic-range capability and stitching algorithm that numerically guarantees pixel accurate whole valve (or any specimen) alignment. It should also be noticed that the presented focusing method is slower compared to contrast methods (appx. 10–20 seconds per focus).

It is known that the porcine aortic valves bear some non-trivial structural differences with human aortic valves. For example, the relative size of the leaflets varies between the species. In porcine aortic valves, the right coronary leaflet has the most surface area and the non-coronary leaflet has the least. In human valves, the non-coronary leaflet has the largest surface area and the left coronary leaflet has the least [12]. Thus, the data presented here may not be directly applicable when considering the structural heterogeneity of the human aortic valves. If sources of human aortic valves are available, our future studies will focus on characterizing the structure of human samples with similar and improved imaging techniques, which in hope will provide direct insights into the design and function of human aortic valves In addition, we are studying the loading dynamic of the fiber bundles and membranes individually, which could integrate with the structural analysis and better understand the structural and mechanical design principles of aortic valves.

### Conclusions

In this study, we applied a novel polarized optical microscopy technique with sub-micron resolution to reveal the mesoscale structural heterogeneity of collagen-fiber based porcine aortic valves. We discovered and quantified the structural heterogeneity between left coronary, non-coronary and right coronary leaflets, at both whole-tissue and fiber bundle levels. Detailed structure of the collagen fiber bundles was revealed, including fiber bundle alignment, branching mechanism and heterogeneity within each of the leaflet. The structure of the less organized membrane and fiber-membrane anchoring mechanism was also investigated. These structural features are suggested to be highly correlated with the biomechanical function of the heart valves. These new insights are valuable for developing more precise models as well as aiding in development of improved, long lasting tissue-engineered, bioprosthetic, and synthetic valve replacements.

## Supporting Information

Figure S1 **Polarized light images of aortic leaflets with different layers on top.** All leaflets from this image came from the same heart. The ventricularis layer images are flipped vertically to ease comparison. We observed only marginal differences in the images.
(TIFF)

Figure S2 **Bar graph of fiber bundle root width** ***W_r_*** **as a function of number of fiber bundles (max of** ***N_r_*** **).** As the number of fiber bundles increase, the mean width decreases.
(TIFF)

Figure S3 **Bar graphs of the length to the first branch point (** ***L_b_*** **) for the for fiber bundles on the left and right sides of each leaflet relative to their position from the free edge (** ***N_r_*** **).** Error bars indicate standard error. While there is no significant difference in the mean distance from leaflet to leaflet or from side to side, the right coronary leaflet’s lower fiber bundles branched at a greater distance from the root than the bundles closer to the free edge. ANOVA1 results: Leaflet *p*>.05, Side *p*>.05, *N_r_ p*<.0001.
(TIFF)
