# Supplementary figures and images for: Complex Collagen Fiber and Membrane Morphologies of the Whole Porcine Aortic Valve
Source: PLoS One. 2014 Jan 21;9(1):e86087. doi: 10.1371/journal.pone.0086087 (PMC3897645; doi:10.1371/journal.pone.0086087)

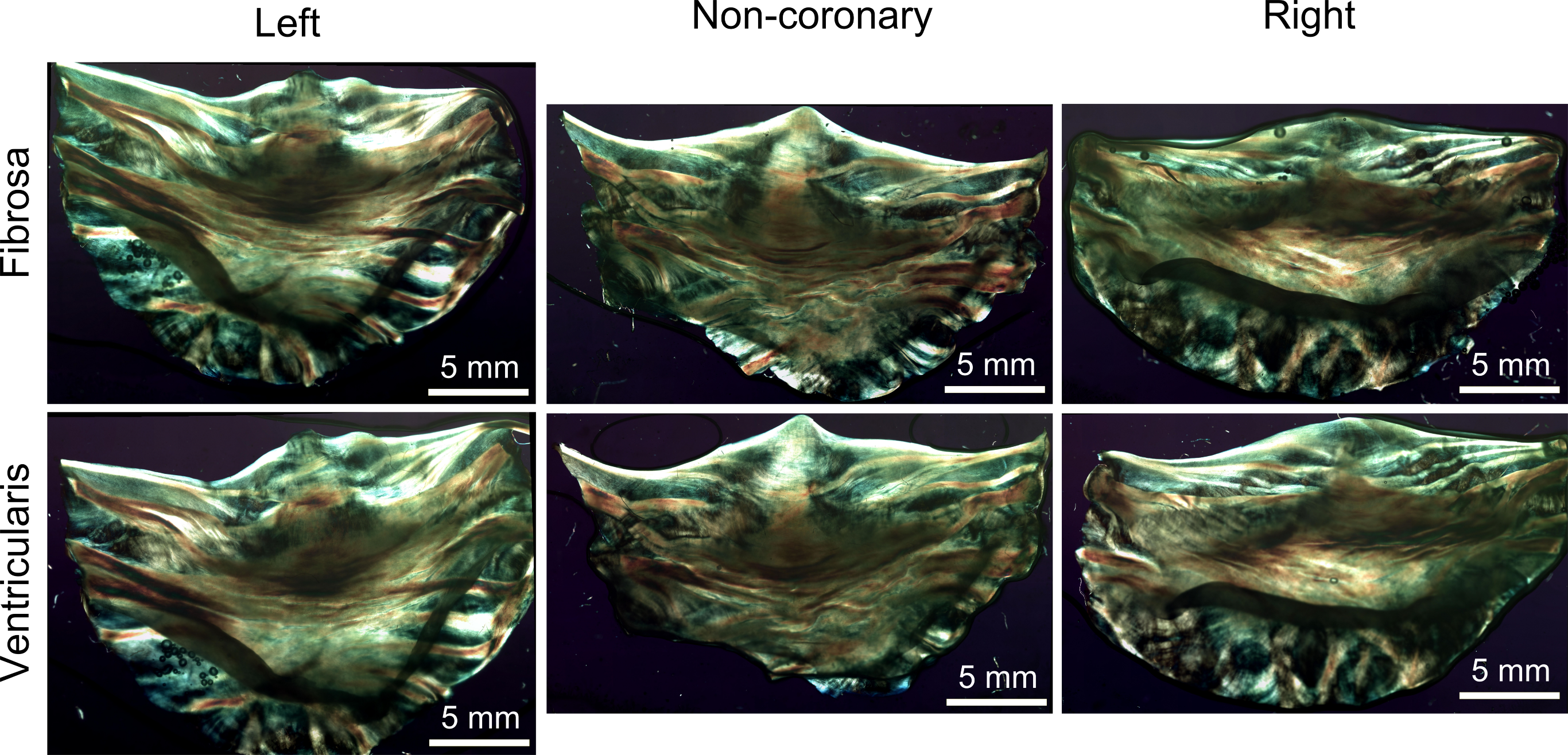

Supplement: Figure S1 — Polarized light images of aortic leaflets with different layers on top. All leaflets from this image came from the same heart. The ventricularis layer images are flipped vertically to ease comparison. We observed only marginal differences in the images. (TIFF) [file pone.0086087.s001.tiff]

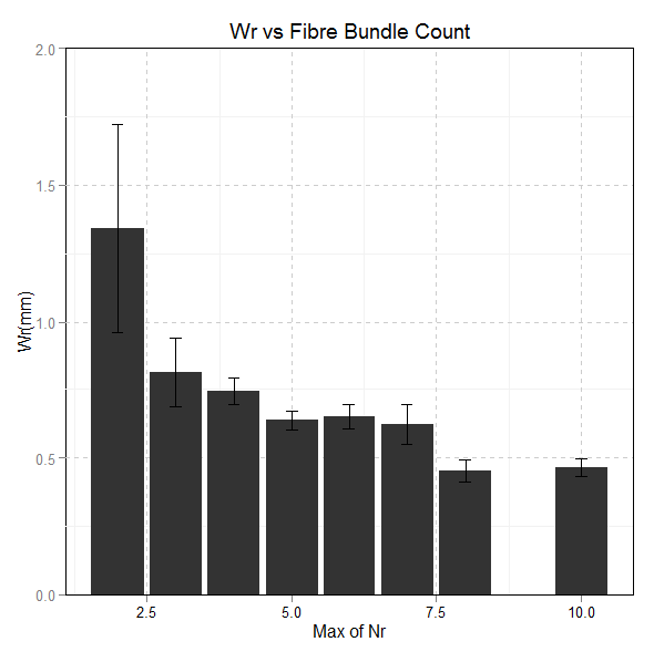

Supplement: Figure S2 — Bar graph of fiber bundle root width Wr as a function of number of fiber bundles (max of Nr ). As the number of fiber bundles increase, the mean width decreases. (TIFF) [file pone.0086087.s002.tiff]

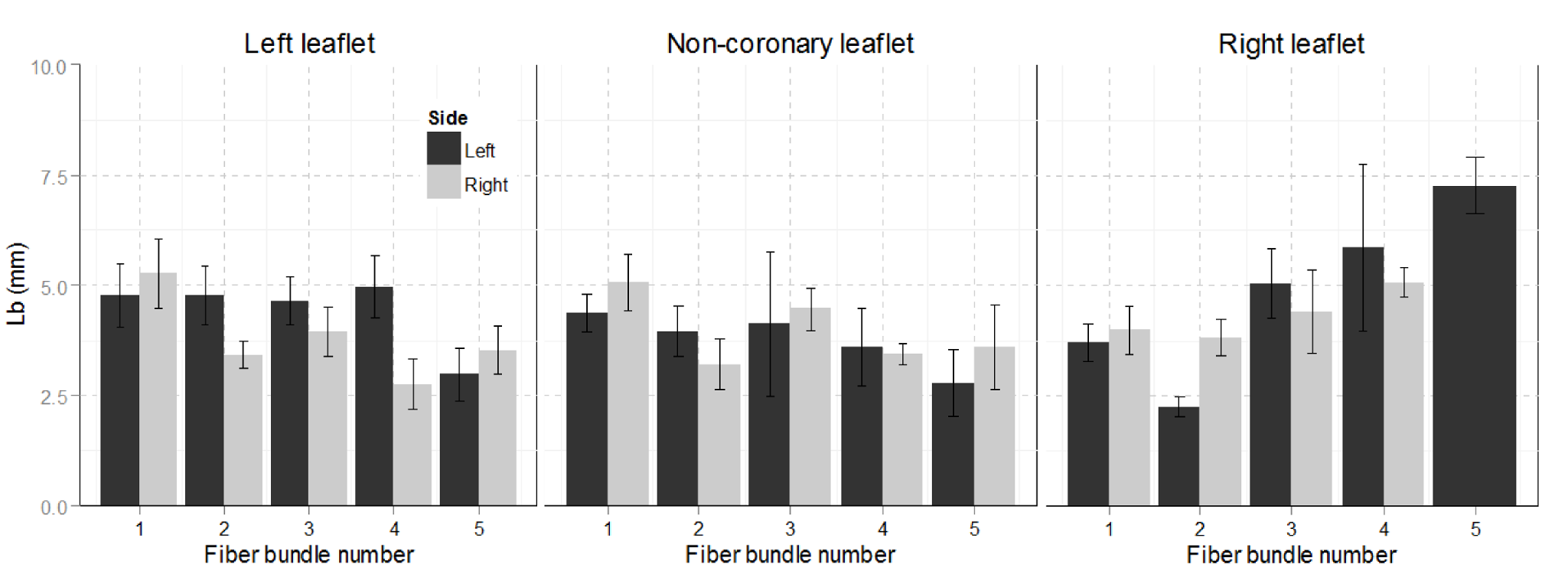

Supplement: Figure S3 — Bar graphs of the length to the first branch point ( Lb ) for the for fiber bundles on the left and right sides of each leaflet relative to their position from the free edge ( Nr ). Error bars indicate standard error. While there is no significant difference in the mean distance from leaflet to leaflet or from side to side, the right coronary leaflet’s lower fiber bundles branched at a greater distance from the root than the bundles closer to the free edge. ANOVA1 results: Leaflet p>.05, Side p>.05, Nr p<.0001. (TIFF) [file pone.0086087.s003.tiff]
